# Cell Partitioning Design for Microfluidic ATPS Devices: A Dynamic Energy Strategy and Calculation Using Chondrocytes and Model Microparticles

**DOI:** 10.3390/mi16080926

**Published:** 2025-08-12

**Authors:** Gabriel Garibaldi, Jimena Alegria, Anita Shayan, Robert Stannert, Nehal I. Abu-Lail, Gongchen Sun

**Affiliations:** Department of Biomedical Engineering and Chemical Engineering, The University of Texas at San Antonio, San Antonio, TX 78249, USA

**Keywords:** Aqueous Two-Phase Systems (ATPS), cell separation, surface energy, microfluidics, human chondrocytes

## Abstract

Sorting and isolating specific cells from heterogeneous populations are crucial for many biomedical applications, including drug discovery and medical diagnostics. Conventional methods such as Fluorescent Activated Cell Sorting (FACS) and Magnetic Activated Cell Sorting (MACS) face limitations in throughput, cost, and the ability to separate subtly different cells. Cell partitioning in Aqueous Two-Phase Systems (ATPSs) offers a biocompatible and cost-effective alternative, particularly when combined with continuous-flow microfluidics. However, it remains challenging to rationally design microfluidic ATPS devices and operation to separate cells with similar origin but different phenotypes. In this paper, using a model ATPS, polyethylene glycol (PEG)—Dextran (Dex) system, and model cells, human chondrocytes (hChs), and carboxylated polystyrene (PS) microparticles, we systematically characterized the material properties affecting cell partitioning in ATPSs, such as surface energies of the solutions and cells and solution viscosities. We developed an energy balance approach between interfacial energy and viscous dissipation to estimate the interface translocation dynamic of cells partitioning into the preferred phase. Combining the experimental measurement and the energy balance model, our calculation reveals that the time required for complete cell partitioning at the ATPS interface can be exploited in microfluidic ATPS devices to separate hChs with different phenotypes (healthy and diseased). We expect our dynamic energy approach to provide a basis and a design strategy for optimizing microfluidic ATPS devices to achieve the efficient separation of phenotypically similar cell populations and further expand the potential of microfluidic cell separation.

## 1. Introduction

Sorting and isolating specific cells or bioparticles from heterogeneous groups is crucial for a variety of applications ranging from disease diagnostics to environmental analysis [1]. Cells possess different physiochemical properties, such as interfacial energy and charge that arise in part from variations in their cytoplasmic structures, membrane compositions, and surface proteins [2]. The differences in cell surface are often taken advantage of to characterize and separate cells, especially benefiting the development of targeted treatments and novel drugs [3].

Several established methods exist for isolating and extracting specific cell types based on their surface differences [4]. Fluorescent Activated Cell Sorting (FACS) uses fluorescent labels to isolate cell populations with specific surface markers [5]; however, its single-cell processing approach makes sorting large samples time-consuming and requires expensive and sophisticated instruments [6]. Magnetic Activated Cell Sorting (MACS) offers faster processing, particularly for large samples, as it handles cells in bulk [7]. Nevertheless, the magnetic beads required for cell surface labeling can be costly, since to remove the magnetic beads, antibodies for those magnetic beads are required, posing a challenge and extra step for large-scale experiments [8]. Density gradient centrifugation effectively separates components based on density [9]; however, this limits its ability to distinguish between cells with similar densities but different physiological states [10].

A promising alternative for cell extraction is phase-based partitioning using Aqueous Two-Phase Systems (ATPSs) [11]. ATPSs involve two incompatible polymer solutions mixed to form an interface. This liquid–liquid phase boundary can serve as a selective filter, allowing certain cells to partition into a distinct phase depending on their surface properties [12]. ATPS-based cell partitioning is biocompatible, economically viable, and easy to implement [13]; it also does not require labeling of cells by fluorescent markers or magnetic beads. Due to these advantages, equilibrium cell partitioning using ATPSs has been proposed and studied since the 1980s. Gerson first showed that the partitioning of lymphocytic cells in a biphasic mixtures of Dextran (Dex) and poly(ethylene glycol) (PEG) could be used to estimate cell surface energy [14]. ATPS-based cell partitioning has also been applied to various fields, such as enabling the separation of specific cell types like immune cells, characterization of conditions such as sickle cell disease, and sophisticated cell patterning where ATPS acts as a “bioink” to create defined cell arrangements or 3D aggregates like spheroids for tissue engineering and drug screening [15,16].

To achieve high-throughput cell partitioning, flow-based microfluidic devices have been developed to implement ATPSs in a continuous laminar flow format while allowing for continuous cell separation. Due to the smaller dimensions in these microfluidic devices, the effects of gravity are minimized, allowing cells to partition to a specific phase based on their properties such as surface wettability instead of density. Several groups have advanced this concept. For example, early work by Yamada et al. demonstrated that a pinched microfluidic channel could improve partitioning efficiency by forcing cells to interact with the ATPS liquid–liquid interface [17]. Soohoo et al. expanded on this strategy and sandwiched a PEG stream with two Dex streams to achieve isolation of leukocytes [18]. Nam et al. reported a similar microfluidic device that achieved 97% efficiency and 100% recovery in separating live and dead CHO-K1 cells with live cells partitioning towards the PEG phase and dead cells being trapped at the PEG-Dex interface [19]. However, none of the existing studies provides a general design method of microfluidic ATPS devices especially for separating live cells with the same origin but different phenotypes.

**Figure 1:**
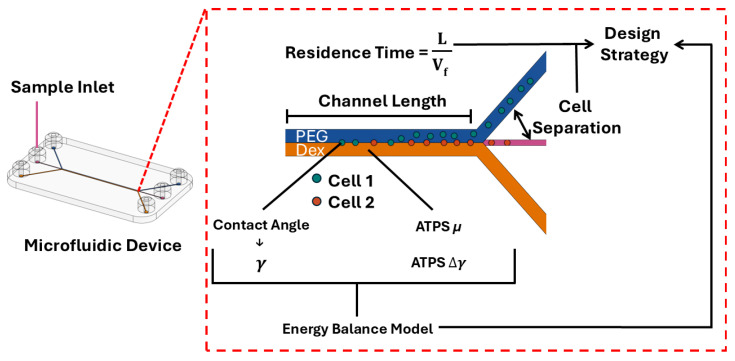
The overview of this study to provide a design strategy for cell separation using a microfluidic ATPS device.

In this paper, we introduce a design strategy for cell partitioning in microfluidic ATPS devices, considering the synergistic effect between preferential partitioning and interfacial translocation dynamic of cells. This strategy is based on the experimentally characterized fundamental material properties of ATPSs and model cells and establishes a dynamic energy model to quantify the interfacial transport of cells. Using PEG and Dex as the model polymer solutions within ATPSs, we systemically measured the surface energy and viscosity of the PEG/Dex ATPS under different concentrations. Using human chondrocytes (hChs) and carboxylated polystyrene (PS) microparticles as models for cells, we experimentally determined the surface energy of different cells. We then developed an energy balance model describing the dynamic of cell preferential partitioning by comparing interfacial energy gradient and viscous energy dissipation. Our model reveals the different phase partitioning behaviors between (1) different types of cells and (2) same type of cells with different physiological states. By relating this dynamic energy approach with microfluidic operation, we aim to provide a novel design strategy (Figure 1) that facilitates the optimization of the system parameters of microfluidic ATPS devices for high-throughput cell separation.

## 2. Materials and Methods

### 2.1. Materials

The following materials were purchased from Sigma Aldrich (St. Louis, MO, USA): fetal bovine serum (FBS), penicillin–strep (pen-strep), dimethyl sulfoxide (DMSO), dextran (Dex) from *Leuconostoc mesenteroides* (molecular weight: 150,000), and poly(ethylene glycol) (PEG) (molecular weight: 35,000). The following materials were purchased from Invitrogen (Carlsbad, CA, USA): Dulbecco’s modified eagle medium (DMEM), phosphate-buffered saline (PBS), and trypan blue. Carboxylated polystyrene (PS) microparticles were purchased from Magsphere (Pasadena, CA, USA).

### 2.2. Pendent Drop Method for Surface Tension Measurements

The total surface tension ($\gamma_{L}$) of aqueous solutions of PEG or Dex at different *w*/*v*% concentrations (*w*/*v*%: 10%, 15%, and 20%) was measured with the pendant drop method at room temperature using a Kruss drop-shape analyzer (model: DSA 100 S, Kruss Scientific, Hamburg, Germany). The pendant drop method determines surface or interfacial tension by analyzing the shape of a liquid drop suspended from a needle, which deforms due to gravity. Using video imaging and numerical fitting of the drop curvature, surface tension is calculated based on density differences and how much the drop deviates from a perfect sphere. A droplet of each liquid was created using a 1 mL syringe and a 1.825 mm outer diameter needle. The analysis is based on the Young–Laplace equation. All measurements were conducted in air as the surrounding phase. Each measurement was performed in triplicate.

### 2.3. Cell Source and Culture

Human chondrocytes (hChs) were donated by StemBioSys, Inc. (San Antonio, TX, USA). Cells were harvested from the knees of 6 male donors, 3 of which were healthy and 3 who were affected by osteoarthritis (OA). The three healthy and diseased donors were 62.3 ± 2.1 and 64.6 ± 1.7 years old, respectively. A statistical analysis was carried out to demonstrate no significant difference in the mean ages between the two groups (*p* = 0.1687). hChs were suspended in preservation medium consisting of culture medium, DMEM, 10% FBS, 1% Pen/Strep, and 1% Amphotericin-B with 10% *v*/*v* DMSO. Following this, the hChs were placed in a freezer to cool at a controlled rate of 1 °C per minute. Cells isolated from each donor were individually stored and labeled with lot numbers, harvest date, and a unique marker to identify the health status of the donor. The cells were then preserved within a cryo-tanks containing liquid nitrogen at a temperature of −180 °C.

To expand cells, cryopreserved vials were thawed at 60 °C in a bead bath, and then centrifuged at 1500 RPM for 10 min. After centrifugation was completed, storage medium containing DMSO was aspirated, leaving the pellet intact. The pellet was resuspended in DMEM/F-12 GlutaMAXTM (L-alanyl-L-glutamine dipeptide). The hChs medium consisted of MEM/F-12, 1% *v*/*v* Pen-Strep, 10% *v*/*v* FBS, and 1% *v*/*v* Fungizone. The hChs culture was incubated at 37 °C, in 5% CO_2_, 95% relative humidity, and 20% oxygen. Cell counting was conducted by using 0.4% trypan blue at a 1:10 dilution. Cells were seeded at a density of 18,000 per mL in 48 well plates sourced from Thermo-Fisher (Waltham, MA, USA). Cultures were conducted in triplicates.

### 2.4. Cell Preparation for Goniometric Analysis

hChs cultures on day 7 were removed from incubation and placed in a biosafety cabinet. Cell culture medium was aspirated and washed with PBS twice. After washing, each of the 48 wells were treated with 400 µL each of collagenase type 1 (0.1% *w*/*v*) for approximately 30 min at 37 °C, in 5% CO_2_, 95% relative humidity, and 20% oxygen. Plates were then placed back in the biosafety cabinet, and each of the 48 wells were micro-pipetted several times to ensure all cells were detached. Cell suspensions were transferred to 2 mL microcentrifuge tubes and centrifuged at 1500 RPM for 10 min. Once complete, the supernatant was aspirated, and intact pellet was left in each microcentrifuge tube. Pellets were resuspended in deionized water (DIW). Filter paper (Whatman 4-micron grade 589/3) was submerged in DIW and stood at room temperature for 5 min before cell suspension was added. The cell suspension was micro-pipetted onto the surface of the filter paper until the suspension was absorbed. The process was repeated until the filter paper’s surface was covered, and a monolayer of cells was achieved. Surface areas for cell coverage were calculated for culture day 7 for cells representing OA and Healthy donors. The filter paper was cut to a circle of 2 cm in diameter and the surface area was calculated. To determine how many cells were needed to form a monolayer on the filter paper, the surface area of the filter paper was divided by the surface area of a hChs cultured for 7 days. This value was multiplied by 5 to ensure that there was a full coverage of cells on the filter paper.

### 2.5. Polystyrene Microparticle Preparation for Goniometric Analysis

**Figure 2:**
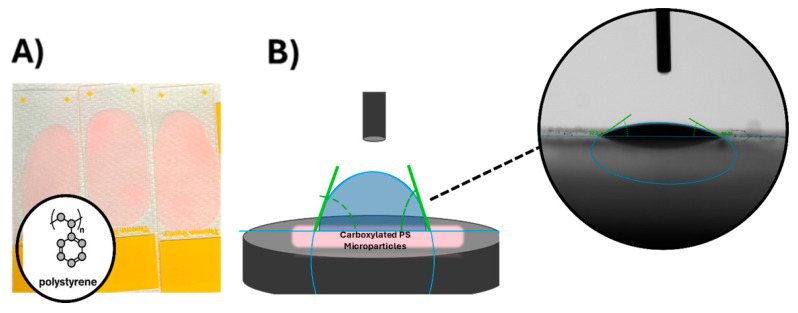
Contact angle measurement of carboxylated PS microparticles and human chondrocytes (hChs). (**A**) Carboxylated PS microparticles on the glass slide (pink areas). (**B**) Schematic illustration of the contact angle measurement setup, and the sessile drop method was used to assess surface wettability of the carboxylated PS microparticle-coated glass slide (or the hChs-coated filter paper). The blue drop illustrated the DIW drop. Experimental contact angle of DIW was determined by the green tangent lines at the solid–liquid–vapor interface.

Carboxylated PS microparticles were placed onto a glass slide for goniometric analysis. These 5.1 µm microparticles were purchased in a 5 mL bottle. The surface area of the glass slide where the particles were placed on was 7 cm × 4 cm, resulting in a total surface area of 28 cm^2^. To achieve a monolayer of carboxylated PS microparticles on the slide, the total surface area of the glass slide was divided by the surface area of one microparticle with a diameter of 5.1 µm. The concentration of carboxylated PS microparticle solution was then calculated to form 5 layers of particles on the glass slide. The solution was micro-pipetted onto the glass slide and left in the vacuum hood overnight to allow for all the excess liquid to evaporate, resulting in a glass slide fully covered by the carboxylated PS microparticles, as depicted in Figure 2A. The complete coverage of carboxylated PS microparticles was validated by atomic force microscopy (Supplemental Materials Section S4).

### 2.6. Goniometric Analysis for Contact Angle Measurements

Contact angles of microparticles and chondrocytes were measured using the sessile drop method with the drop-shaped analyzer DSA100 (KRŰSS Hamburg Germany). The measurements were performed at room temperature and ambient humidity. Contact angle measurements were conducted using DIW for hChs of healthy and OA origins and carboxylated PS microparticles, as depicted in Figure 2B. Each drop of DIW used in measurements was 2 µL in volume. The diameter of this liquid drop using a volume of a sphere was 1.56 mm. Nine contact angles were recorded on each desired surface. The measurements were carried out on at least three different surfaces (Healthy hChs, OA hChs, and carboxylated PS microparticles), and the average contact angle for each material was calculated.

### 2.7. Dynamic Viscosity Measurement of Polymer Solutions

The dynamic viscosity of PEG or Dex solutions with varying *w*/*v*% concentrations was measured using a Brookfield Viscometer from AMATEK Brookfield (Middleborough, MA, USA) equipped with a rotating spindle. For each solution, we performed nine measurements by increasing the spin rate from 20 rpm to 100 rpm in 10 rpm increments. The final viscosity of each solution was the average of nine readings. All measurements have a reported instrument error of less than 10%.

## 3. Results and Discussion

The critical dynamic process of preferential partitioning of cells in a microfluidic ATPS device is the translocation of cells across the interface between two polymer solutions, a phenomenon driven by the interfacial energy gradient and damped by viscous dissipation. To understand this interface translocation dynamic, we systematically characterized the material properties of the ATPS polymer solutions and two distinct cell models: standardized carboxylated PS microparticles and clinically relevant primary human chondrocytes. Using this experimental data, we analyzed the translocation dynamics by an energy balance approach. This method compares the interfacial energy driving a cell to enter its preferred phase with the viscous energy dissipation damping its movement, which reveals unique phase-partitioning behaviors of different cell models and offers insights to rationally design microfluidic ATPS devices for precise cell separation.

### 3.1. Surface Energy of ATPS Solutions

**Figure 3:**
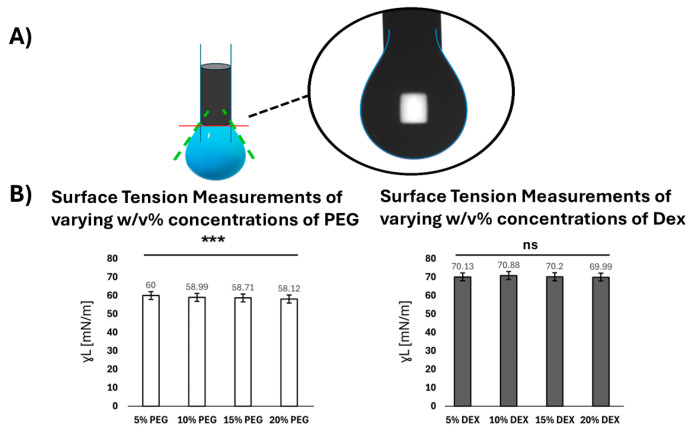
Surface tension measurement of PEG or Dex polymer solutions. (**A**) Schematic and a representative image of a pendant drop. The curvature of the drop is extracted from the blue line. (**B**) Surface tension of PEG solutions with different *w*/*v*% concentrations. Surface tension of Dex solutions with different *w*/*v*% concentrations. A one-way ANOVA confirmed a statistically significant decrease in PEG surface tension across concentrations (*p* < 0.0001), while Dex surface tension showed no significant change (*p* > 0.05, one-way ANOVA). The triple asterisk (***) indicates a statistically significant result at the *p* < 0.001 level. ns indicates that there is no statistically significant difference across different concentrations of Dex.

To understand the interfacial energy between cell models and ATPS solutions, we first sought to measure the surface tension (i.e., surface energy) of the ATPS solutions (PEG or Dex) at different concentrations, using the pendant drop method (Figure 3A). The solution surface tension was determined using droplet shape analysis by the Young–Laplace equation.

As shown in Figure 3B, the surface tensions of PEG solutions are lower than the surface tension of water (72 mN/m). The increase in PEG concentration only slightly decreases the solution surface tension, with values of 60.00 ± 0.02 (5% PEG), 58.99 ± 0.12 (10% PEG), 58.71 ± 0.20 (15% PEG), and 58.12 ± 0.01 mN/m (20% PEG). A one-way ANOVA confirmed this statistically significant decrease in PEG surface tension across concentrations (*p* < 0.0001). The ANOVA calculation is based on the ratio of variance between groups to variance within groups. Based on the literature, the surface tensions of pure PEG-based Deep Eutectic Solvents (DESs), reported by Chen et al. [20], are around 44–45 mN/m; and the addition of water could increase the PEG solution surface tension to approximately 60 mN/m. These results agree with the surface tension behavior observed in our PEG solution, where the higher water content correlates with a slightly higher surface tension. The surface tensions of the Dex solutions on the other hand, are close to the surface tension of water and show no significant variation with the increase in concentration, with values recorded as 70.13 ± 0.05 (5% Dex), 70.88 ± 0.05 (10% Dex), 70.20 ± 0.03 (15% Dex), and 69.99 ± 0.06 mN/m (20% Dex). A one-way ANOVA further confirmed no statistically significant difference in Dex surface tension across concentrations (*p* > 0.05). We compared our results with the literature data reported by Hoorfar et al. [21]. The surface tension of Dex solutions (Mw = 68,800) at 24 °C and at concentrations of 0–20 mg/mL decreased slightly from 72.26 ± 0.01 mN/m (pure water) to 71.26 ± 0.01 mN/m (20 mg/mL Dex solution) [21]. The Dex solutions measured in our work present similar values and are only slightly lower than those reported by Hoorfar et al. [20], possibly due to the higher Dex concentration and molecular weight used in our experiment.

### 3.2. Dynamic Viscosity of ATPS Solutions

**Figure 4:**
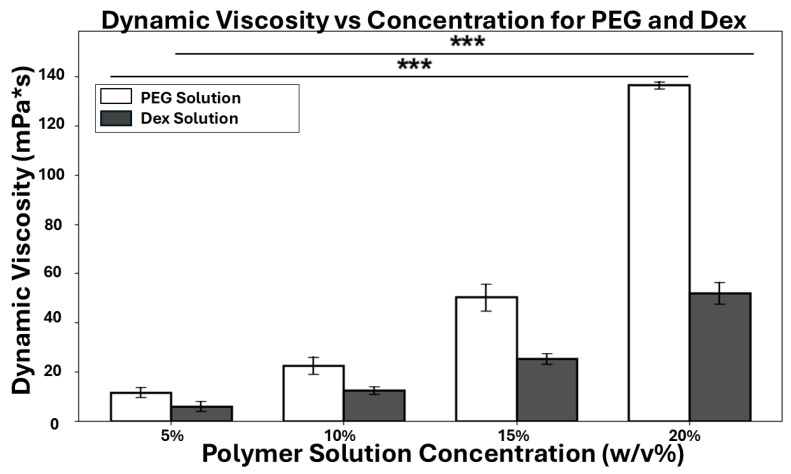
The dynamic viscosities of PEG and Dex solutions with increasing polymer concentrations. One-way ANOVA tests confirmed statistically significant increases in dynamic viscosity across concentrations for both PEG (*p* < 0.0001) and Dex (*p* < 0.0001) solutions. The triple asterisk (***) indicates a statistically significant result at the *p* < 0.001 level.

As particles or cells partition into their preferred phase, they will experience a drag force from the surrounding fluid that damps their movement. To study the dynamic process of the preferential partitioning, we measured the dynamic viscosities of ATPS solutions with different concentrations. As shown in Figure 4, the magnitude of the solution viscosity is directly related to the polymer concentration; as the *w*/*v*% of PEG or Dex increases, a greater polymer chain entanglement and friction leads to a higher dynamic viscosity. One-way ANOVA tests confirmed statistically significant increases in dynamic viscosity across concentrations for both PEG (*p* < 0.0001) and Dex (*p* < 0.0001) solutions. A four-fold increase in polymer concentration can result in an order of magnitude higher in dynamic viscosity. At the same concentration, the PEG solution is about as twice viscous as the Dex solution. We will explore these fluid properties to understand the particle/cell partitioning dynamic in later sections.

### 3.3. Contact Angle of Cell Models

**Figure 5:**
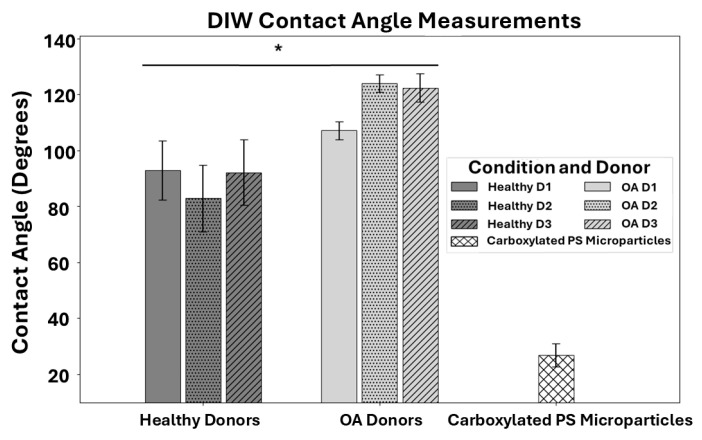
DIW contact angle measurements were used to assess surface properties of hChs derived from healthy and osteoarthritic (OA) donors after 7 days of culture and carboxylated PS microparticles. Each bar represents the mean of 9 DIW contact angles measured on triplicate cultures for each donor, with error bars indicating standard deviation. A Linear Mixed Model (LMM) analysis indicated that OA hChs exhibited a significantly higher contact angle compared to healthy hChs (*p* = 0.036). The asterisk (*) in the figure indicates a statistically significant difference between the “Healthy Donors” and “OA Donors” groups at the *p* < 0.05 level.

To characterize the surface properties of cell models, we measured the contact angle of DIW on hChs or carboxylated PS microparticles. Goniometric measurements were performed on a layer of hChs derived from OA and healthy donors as well as a layer of carboxylated PS microparticles as a reference. Cells were cultured for seven days prior to conducting the goniometric analysis. In this study, we primarily focused on the day 7 timepoint since it captures a crucial balance where the cells have adapted to the culture to produce significant, measurable levels of glycosaminoglycan (GAG) and collagen and exhibit peak viability and proliferation, yet are only in the early stages of differentiation. The DIW contact angles measured on hChs isolated from healthy and OA donors as well as those for the carboxylated PS microparticles are shown in Figure 5. The contact angles of hChs from healthy donors are on average less than 90 degrees as opposed to the ones of hChs from OA donors, being close to 120 degrees on average, highlighting the difference in wettability between the two groups. The PS microparticles on average expressed a much lower contact angle due to them being carboxylated. When compared to a DIW contact angle of 65°, the threshold DIW contact angle of a hydrophilic surface [22], all cells are hydrophobic as expected for hChs [23], and the carboxylated PS microparticles are hydrophilic. When donor to donor variations were compared, slight differences between the DIW contact angles amongst donors were observed. However, there are no significant statistical differences from donor to donor for both the healthy and OA groups. To account for inherent biological variability, a Linear Mixed Model (LMM) was utilized to analyze the differences observed in the hChs contact angle data (Figure 5). LMMs are particularly advantageous over traditional ANOVA for data that includes repeated measures or observations nested within groups, such as individual donors. The LMM analysis allows us to model ‘Donor’ as a random effect, thereby accounting for unmeasured donor-specific factors (e.g., diet, activity levels, or other physiological differences) that might introduce correlation among measurements from the same individual. Thus, the LMM provides more accurate and robust estimates of the fixed effects (e.g., the influence of disease status) and their statistical significance (see details in Supplemental Materials Section S5). The LMM revealed a statistically significant overall effect of ‘Disease’ status on cell surface properties, indicating that OA hChs exhibited a significantly higher contact angle (estimated 9.98 degrees higher) compared to healthy hChs (*p* = 0.036). Specific DIW contact angle measurements are reported in Supplemental Materials (Table S1).

### 3.4. Interfacial Energy Between Cells and ATPS Solutions

Based on the surface properties of cell models and ATPS solutions, we sought to calculate the interfacial energy between the cell models and various ATPS solutions, which will determine the preferred phase of cell models and help understand their partitioning dynamics. First, we numerically calculated the total surface energy of hChs models and carboxylated PS microparticles (Table 1) using the measured DIW contact angle ($\theta_{DIW}$) and Neumann’s equation of state for interfacial tensions of solid–liquid systems (Equation (1)) [24]:

(1) $$\frac{\left(\cos\theta_{DIW}+1\right)}{2}=\sqrt{\frac{\gamma_{S:cell}}{\gamma_{L:DIW}}}e^{\left[-\beta {\left(\gamma_{L:DIW}-\gamma_{S:cell}\right)}^{2}\right]}$$

where $\gamma_{S:cell}$ represents the total surface energy of hChs or carboxylated PS microparticles (solid phase) to be solved; $\gamma_{L:DIW}$ is the total surface energy of DIW (liquid phase); and $\theta_{DIW}$ is the measured contact angle of DIW on cell models. **β** is a universal empirical constant with a value of 0.000115 (m^2^/mJ)^2^, validated by Neumann and colleagues for low-energy, non-metallic solids [24,25,26].

Next, we applied Neumann’s equation of state again to predict the theoretical wettability of various PEG or Dex solutions on the cell models:

(2) $$\frac{\left(\cos\theta_{PEG/Dex-cell}+1\right)}{2}=\sqrt{\frac{\gamma_{S:cell}}{\gamma_{L:PEG/Dex}}}e^{\left[-\beta {\left(\gamma_{L:PEG/Dex}-\gamma_{S:cell}\right)}^{2}\right]}$$

where $\left(cos\theta_{PEG/Dex-cell}+1\right)$ is the wettability factor of the ATPS solution (PEG or Dex solution) on cell models. To predict $\left(cos\theta_{PEG/Dex-cell}+1\right)$, we used the calculated total surface energy of cell models ($\gamma_{S:cell}$, solid phase) and the measured total surface energy (i.e., surface tension) of the ATPS solution ($\gamma_{L:PEG/Dex}$, liquid phase).

Finally, using the predicted wettability factor ($cos\theta_{PEG/Dex-cell}+1$) and the total surface energies of the solid phase (cell models, $\gamma_{S:cell}$) and liquid phase (ATPS solutions, $\gamma_{L:PEG/Dex}$), we calculated the interfacial energy between cell models and various ATPS solutions ($\gamma_{PEG/Dex-cell}$) using Young’s Equation:

(3) $$\gamma_{PEG/Dex-cell}={\gamma_{L:PEG/Dex}+\gamma }_{S:cell}-(\cos\theta_{PEG/Dex-cell}+1)\cdot \gamma_{L:PEG/Dex}$$

The calculated interfacial energy between different ATPS solutions (PEG or Dex solutions with different concentrations) and various cell models (Healthy and OA hChs and carboxylated PS microparticles) is summarized in Table 2.

**Figure 6:**
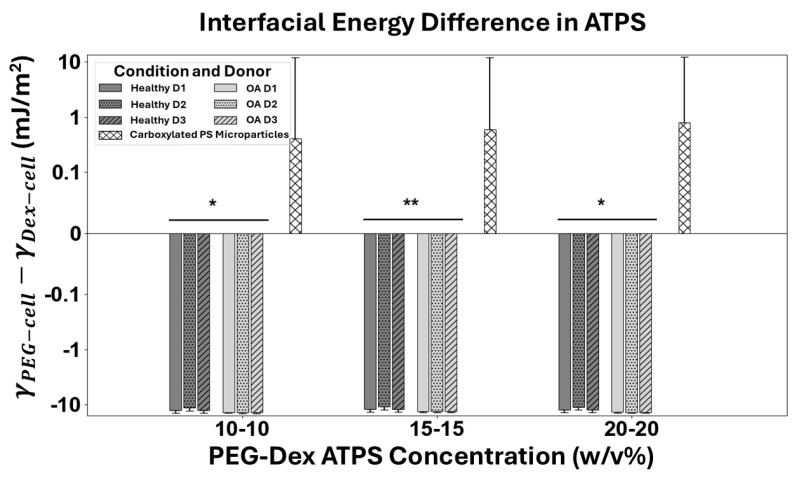
The difference in interfacial energy between $\gamma_{Dex-cell}$ and $\gamma_{PEG-cell}$ for healthy and OA hChs and carboxylated PS microparticles. Carboxylated PS microparticles have lower interfacial energy in Dex as opposed to hChs, which have a lower interfacial energy in PEG, indicating that hChs has a strong preference to partition in the PEG phase, while carboxylated PS microparticles prefer the Dex phase. Error bars represent the propagated uncertainty of its calculated interfacial energy difference. Statistical analysis (independent *t*-tests) confirmed significant distinctions between healthy and OA groups across all concentrations (10-10 Concentration: *p* = 0.0163; 15-15 Concentration: *p* = 0.0094; and 20-20 Concentration: *p* = 0.0135). The asterisk (*) indicates a statistically significant difference between the Healthy and OA groups at the *p* < 0.05 level, and the double asterisk (**) indicates a statistically significant result at the *p* < 0.01 level.

To determine the preferred phase of cell models in the ATPS, we examined the difference in interfacial energies of cell models between the two phases ($\gamma_{PEG-cell}-\gamma_{Dex-cell}$) in ATPSs with varying concentrations: 10 *w*/*v*% PEG/10 *w*/*v*% Dex, 15 *w*/*v*% PEG/15 *w*/*v*% Dex, and 20 *w*/*v*% PEG/20 *w*/*v*% Dex. As shown in Figure 6, we found that carboxylated PS microparticles, as a model for general hydrophilic cells [27], prefer the Dex phase since the total interfacial energy of carboxylated PS microparticles is smaller in the Dex phase. This preference is also experimentally validated by the prior literature [27]. On the other hand, hChs prefer the PEG phase due to their smaller total interfacial energy in the PEG solution, which is consistent with the knowledge that chondrocytes are mostly hydrophobic [23]. hChs can hence be separated from hydrophilic cells in ATPSs simply by preferred partitioning. However, the interfacial energy differences between healthy and OA hChs groups were similar, and they both preferred the PEG phase. Therefore, it is challenging to separate healthy hChs from OA hChs by simple equilibrium-based ATPS phase partitioning. A different dynamic strategy is needed.

### 3.5. Interface Translocation Dynamics of hChs

Lastly, we sought to design a strategy to separate different hChs using microfluidic ATPS devices by understanding the interface translocation dynamics of hChs. To partition into the preferred phase, cells need to transport across the interface. We argue that the time required for different cells to fully translocate from the interface into the preferred phase could serve as the basis for separation in a continuous-flow microfluidic device. Therefore, we analyzed the interface translocation dynamics of hChs via an energy balance approach.

The translocation of a cell into the preferred phase is driven by the interfacial energy difference:

(4) $$E_{driving}=2\pi R^{2}\Delta \gamma =2\pi R^{2}\left|\gamma_{PEG-cell}-\gamma_{Dex-cell}\right|$$

where R is the radius of the cell.

This driving energy will be balanced by the viscous dissipation due to Stokes drag:

(5) $$E_{dissipation}=6\pi R\mu_{avg}v\times 2R$$

where we approximate that the cell transports across the interface with a constant terminal velocity v. This assumption is supported by Singh et. al.’s study on bubble dynamics in viscous immiscible liquids, which shows that, once interfacial deformation stabilizes, microscale bubbles’ ascent through viscous immiscible liquids proceeds at a nearly steady velocity [28], and by Sinha et al.’s observations of microspheres “snapping in” and traversing the interface with minimal acceleration [29]. To fully translocate into the preferred phase from the interface, the cell must travel at least a full diameter distance, 2R; hence, the viscous energy dissipation, $E_{dissipation}$, is the work conducted by the Stokes drag force over the full translocation distance. To account for the different viscosities of two phases on the two sides of the ATPS interface, we used the average viscosity, μavg=(μPEG+μDex)2, to approximate the viscous energy dissipation.

By balancing the driving energy and the dissipation energy, we can calculate the terminal velocity of the cell: v=Δγ6μavg. Therefore, the time required for a cell to fully translocate into the preferred phase can be defined by 2Rv, as follows:

(6) $$\tau_{p}=\frac{12\mu_{avg}R}{\Delta \gamma }$$

**Figure 7:**
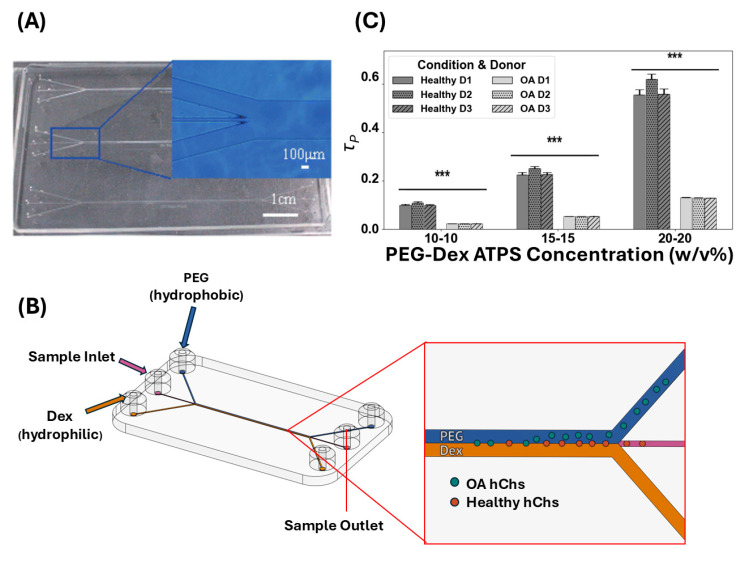
The image and schematics of a microfluidic ATPS device and the $\tau_{p}$ of healthy and OA hChs in PEG-Dex ATPSs with different concentrations. (**A**) Image of the actual microfluidic device used by Tsukamoto et al. to separate leukocytes, erythrocytes, and amphipathic cells with text enlarged, adapted from Ref. [30]. (**B**) Schematic of a microfluidic ATPS device, inspired by Tsukamoto et al. [30]. (**C**) $\tau_{p}$ of healthy and OA hChs in PEG-Dex ATPS with different concentrations. Error bars represent the standard error of the mean (SEM), with values of 0.0036 ms, 0.0084 ms, and 0.0210 ms for healthy chondrocytes at 10-10%, 15-15%, and 20-20% concentrations, respectively, and 0.0001 ms for OA chondrocytes across all three concentrations. The differences in $\tau_{p}$ between healthy and OA hChs was found statistically significant at all concentrations (*p* = 0.0001 for all comparisons) using independent *t*-tests. The triple asterisk (***) indicates a statistically significant result at the *p* < 0.001 level.

Equation (6) shows that the partitioning time of cells, $\tau_{p}$, is determined by not only the interfacial energy difference, but also the cell size (for further details, see Supplemental Materials Section S2) and the viscosities of ATPS solutions (measured in Section 3.2). By calculating the $\tau_{p}$ of hChs from healthy or OA donors in ATPSs with different concentrations, we found that there is a significant difference between the healthy and OA hChs phenotypes. Statistical analysis (independent *t*-tests) confirmed that the difference in partitioning time ($\tau_{p}$) between healthy and OA hChs was statistically significant at all concentrations (*p* = 0.0001 for all comparisons), with healthy hChs consistently exhibiting higher $\tau_{p}$ values. The $\tau_{p}$ for healthy hChs is consistently higher (3–5-fold) than the $\tau_{p}$ for OA hChs for every PEG-Dex concentration pair and amongst every donor for each condition. In addition, the higher the PEG-Dex concentration, the larger the difference in $\tau_{p}$ between OA and healthy hChs, as shown in Figure 7. This is due to the amplification of $\tau_{p}$ by the viscosity of the ATPS solutions. Specific partitioning times are summarized in Supplemental Materials (Table S3). The $\tau_{p}$ dependence on the concentration underscores a potential opportunity for optimizing the ATPS. By carefully selecting PEG and Dex concentrations, one can fine-tune both the driving force (Δγ) and the viscous resistance ($\mu_{avg}$), thereby controlling the partitioning time ($\tau_{p}$) and maximizing the separation efficiency between different cell populations. While higher concentrations amplify the $\tau_{p}$ difference, practical considerations such as solution stability and biocompatibility should also be considered to determine the optimal concentration range for a certain application.

Microfluidic ATPS devices can exploit this difference in $\tau_{p}$ to separate cells with similar origin but different phenotypes. Under a continuous-flow condition, cells have a limited residence time inside the device. This residence time can be designed between the $\tau_{p}$ of two different cells (e.g., healthy and OA hChs) such that cells with a shorter $\tau_{p}$ can migrate from the ATPS interface fully into the preferred phase (i.e., PEG phase for hChs), while other cells remain bound at the interface when exiting the device. Assuming cells move at the same flow velocity as the fluid, the quantitative relationship between microfluidic channel’s physical dimensions, flow rate, and the cell residence time can be approximated as follows:

(7) $$Residence\;Time\;\approx \;\frac{Channel\;Length\;\times \;Cross\;-\;sectional\;Area}{Flow\;Rate}$$

We validated this analysis by comparing it with the prior literature. Tsukamoto et al. reported a microfluidic ATPS device, as depicted in Figure 7, to separate erythrocytes from Jurkat cells [30]. The microchannel has a depth of 100 µm, a width of 30 µm, and a channel length of 5 cm with a flow rate of 0.1 to 3 mL/min. Using the same microfluidic ATPS device, only by changing the channel length to 1 cm and using a flow rate of 4.5 mL/min, one could create a cell residence time of 0.4 ms and separate OA and healthy hChs with our dynamic strategy. Such a microfluidic device could be readily fabricated with current manufacturing technology, and the flow rate is easily achievable with a syringe pump.

## 4. Limitations and Future Studies

Although cells in this study were not air-dried but instead filtered onto paper immediately after removal from the flask, and contact angle measurements were taken shortly thereafter, it is possible that this preparation could partially alter the native surface properties of live, suspended cells. Mege et al. demonstrated that contact angles are influenced by the drying procedure, with even moderate changes in humidity altering apparent hydrophobicity, suggesting that any deviation from physiological hydration, including filtration, may affect surface energy [31]. Majhy et al. further showed that mammalian cell adhesion and proliferation are highly sensitive to substrate surface energy under hydrated conditions, with optimal behavior observed on moderately hydrophilic surfaces (~70 mJ/m^2^) [32]. While our filtering protocol does preserve viability, it does not guarantee that surface molecular architecture remains fully unaltered. Future studies should incorporate real-time surface energy measurements on live, suspended cells in aqueous environments—such as using force spectroscopy or dynamic wettability assays—to better capture physiologically accurate surface properties.

Moreover, while our energy balance model assumes constant terminal velocity for hChs across the interface, direct experimental validation, although beyond the scope of this study, could offer crucial insights to refine the model and understanding of interfacial cell transport in the microfluidic ATPS. Dynamic imaging techniques, such as micro-particle image velocimetry, could be used in future works for experimental validation.

## 5. Conclusions

This study successfully developed a dynamic strategy to design determining optimal microfluidic ATPS devices for cell separation applications. We systematically measured the fundamental material parameters governing the cell partitioning in ATPSs, including the surface energy of ATPS solutions and cells and the viscosities of ATPS solutions. Using model ATPS (PEG-Dex system) and cell models (hChs and carboxylated PS microparticles), our energy calculation correctly predicted the preferential partitioning of hydrophilic carboxylated PS microparticles into the Dextran phase and hydrophobic hChs into the PEG phase based on minimization of interfacial energy. To expand the separation power of microfluidic ATPS devices, we used an energy balance approach to understand the dynamic of cell partitioning across the ATPS interface and found a distinct difference in partitioning times between healthy and OA hChs, cells difficult to separate using equilibrium-based phase partitioning. A new design strategy is proposed to use continuous-flow microfluidic ATPS device to separate cells with similar origin but different phenotypes and validated by comparing with the existing literature. This strategy provides a new perspective for rationally designing microfluidic ATPS devices. By predicting the partitioning time based on measurable physicochemical properties, the ATPS solution parameters (e.g., polymer concentrations and molecular weights) along with device parameters (e.g., channel size and flow rate) can be optimized to achieve efficient, high-throughput separation of specific cell populations, including those with subtle phenotypic variations like healthy versus diseased states. We expect this work to further harness the potential of microfluidic ATPS devices for various cell sorting and separation applications.

## Figures and Tables

**Table 1:** Solid surface energy.

Group	Surface Energy (mJ/m^2^)
Healthy hChs Donor 1	26.75
Healthy hChs Donor 2	33.06
Healthy hChs Donor 3	27.22
OA hChs Donor 1	18.06
OA hChs Donor 2	9.01
OA hChs Donor 3	9.78
Carboxylated PS Microparticles	65.93

**Table 2:** Interfacial energies between polymer solutions and cell models and standard deviation between donor variability (hChs and carboxylated PS microparticles) (mJ/m^2^).

Group	Concentration (*w*/*v*%)	Average	Standard Deviation
PEG—Healthy	10%	13.48	2.67
	15%	13.23	2.60
	20%	12.72	2.56
PEG—OA	10%	29.67	5.67
	15%	29.37	5.58
	20%	28.73	5.45
Dex—Healthy	10%	25.79	3.46
	15%	24.99	3.46
	20%	24.74	3.45
Dex—OA	10%	43.58	5.71
	15%	42.74	5.70
	20%	42.48	5.69
PEG—Carboxylated PS Microparticle	10%	0.88	
	15%	0.95	
	20%	1.11	
Dex—Carboxylated PS Microparticle	10%	0.48	
	15%	0.35	
	20%	0.32	

## Data Availability

The original contributions presented in this study are included in the article/Supplementary Material. Further inquiries can be directed to the corresponding author.

## Supplementary Materials

The following supporting information can be downloaded at: https://www.mdpi.com/article/10.3390/mi16080926/s1. Table S1: Summary of deionized water contact angle measurements; Table S2: Size data of chondrocytes and their calculated diameters (μm); Table S3: Summary of partitioning times; Figure S1: Schematic of AFM setup; Figure S2: Carboxylated PS microparticle separation analysis; Table S4: Model fit summary; Table S5: Fixed-effects omnibus tests; Table S6: Parameter estimates (fixed coefficients); Table S7: Random components.

## Abbreviations

The following abbreviations are used in this manuscript:

PEG	Polyethylene Glycol
Dex	Dextran
PS	Polystyrene
AFM	Atomic Force Microscope
ATPS	Aqueous Two-Phase System
MACS	Magnetic Activated Cell Sorting
FACS	Fluorescent Activated Cell Sorting
OA	Osteoarthritis
DIW	Deionized Water
GAG	Glycosaminoglycan
DMEM	Dulbecco’s Modified Eagle Medium
PBS	Phosphate-Buffered Saline
hChs	Human Chondrocytes
LMM	Linear Mixed Model
**τ_p_**	Partitioning Time
