# Supplementary material for: Cell Partitioning Design for Microfluidic ATPS Devices: A Dynamic Energy Strategy and Calculation Using Chondrocytes and Model Microparticles
Source: Micromachines (Basel). 2025 Aug 12;16(8):926. doi: 10.3390/mi16080926 (PMC12388751; doi:10.3390/mi16080926)
Supplement: Supplementary file 1 [file micromachines-16-00926-s001.zip › micromachines-3792617-supplementary.pdf]

# Supplementary Materials

## Design Cell Partitioning in Microfluidic ATPS devices: A Dynamic Energy Strategy and Calculation Using Chondrocytes and Model Microparticles

**Gabriel Garibaldi [1,2], Jimena Alegria [1,2,&], Anita Shayan [1,2,&], Robert Stannert[1,2], Nehal Abu-Lail[1,2], and Gongchen Sun[1,2,*]**

[1] Affiliation 1-Department of Biomedical Engineering and Chemical Engineering, The University of Texas at San Antonio, San Antonio, Texas, 78249
[2] Affiliation 2- The University of Texas Health at San Antonio, San Antonio, Texas, 78229
* Correspondence: Gongchen.sun@utsa.edu; 210-458-7919.
& authors contributed equally to the paper

## Supplementary Text

### S1. Deionized Water and Polymer Solution Contact Angle Measurements

Contact angle measurements were made on different conditions of human chondrocytes (hChs) and carboxylated polystyrene (PS) microparticles using Deionized Water (DIW) as the solvent. The different conditions of hChs were Healthy groups and Osteoarthritis (OA) groups and there was a total of 6 donors, 3 for each condition. Day 7 hChs were used because they express peak proliferation and viability at this point. hChs and carboxylated PS microparticles were immobilized on glass substrates, and the contact angles were measured for all groups depicted on Table S1.

**Table S1. Summary of Deionized Water Contact Angle Measurements**

| Donor | Healthy Donor Chondrocytes D7 (°) | OA Donor Chondrocytes D7 (°) | Carboxylated PS Microparticles |
|---|---|---|---|
| **N/A** | | | 26.76 ± 4.19 |
| **1** | 92.86 ± 10.58 | 107.10 ± 3.27 | |
| **2** | 82.88 ± 11.94 | 124.01 ± 3.06 | |
| **3** | 92.12 ± 11.64 | 122.39 ± 5.06 | |

## S2. Average Volume of hChs

hChs isolated from healthy and OA donors were imaged using AFM. The dimensions (length, width, and height) of the hChs derived from healthy and OA donors were measured from at least three images per phenotype. The height of the hChs was measured using AFM. The average volume (μm$^3$) of the hChs at day 7 was calculated using the measured dimensions by assuming an elliptical cell geometry:

$$V = \frac{4}{3}\pi abc \quad (1)$$

In this context, a, b, and c represent the radial height, length, and width of the hChs, respectively. Approximating ellipsoidal cells as spheres is a valid simplification that makes the physics mathematically manageable, as the cells' near-spherical shape means the model accurately represents the average behavior of a large, randomly oriented population. Therefore, using the volume of the hChs at day 7 we calculated representative diameters for Healthy and OA hChs as shown in Table S2:

$$D = \sqrt[3]{\frac{6V}{\pi}} \quad (2)$$

**Table S2. Size Data of Chondrocytes and their Calculated Diameters (μm)**

| hChs Condition | Length | Width | Height | Volume | Diameter |
|---|---|---|---|---|---|
| **Healthy** | 20.34 | 30.03 | 3.01 | 963 | 12.25 |
| **OA** | 34.35 | 18.25 | 3.23 | 1060 | 12.65 |

## S3. Human Chondrocyte/Carboxylated PS Microparticle Partitioning Time in ATPS

**Table S3. Summary of Partitioning Times**

| PEG-Dex_Pair (w/v%) | Donor | Partition Time (ms) |
|---|---|---|
| | **Carboxylated PS Microparticles** | |
| **10-10** | N/A | 1.342 |
| **15-15** | N/A | 1.973 |
| **20-20** | N/A | 3.752 |
| | **Healthy** | |
| **10-10** | 1 | 0.099 ± 0.003 |
| **15-15** | 1 | 0.226 ± 0.008 |
| **20-20** | 1 | 0.555 ± 0.020 |
| **10-10** | 2 | 0.110 ± 0.003 |
| **15-15** | 2 | 0.251 ± 0.008 |
| **20-20** | 2 | 0.619 ± 0.020 |
| **10-10** | 3 | 0.100 ± 0.003 |
| **15-15** | 3 | 0.227 ± 0.008 |
| **20-20** | 3 | 0.558 ± 0.020 |
| | **OA** | |
| **10-10** | 1 | 0.024 ± 0.000 |
| **15-15** | 1 | 0.054 ± 0.000 |

| | | |
|---|---|---|
| **20-20** | 1 | 0.132 ± 0.000 |
| **10-10** | 2 | 0.024 ± 0.000 |
| **15-15** | 2 | 0.053 ± 0.000 |
| **20-20** | 2 | 0.130 ± 0.000 |
| **10-10** | 3 | 0.023 ± 0.000 |
| **15-15** | 3 | 0.053 ± 0.000 |
| **20-20** | 3 | 0.129 ± 0.000 |

## S4. Atomic Force Microscopy to Validate Microparticle Preparation for Goniometric Analysis

Carboxylated PS microparticles were immobilized overnight on a glass slide for Atomic Force Microscopy (AFM) analysis. To ensure at least a monolayer coverage on the slide, a 111.7 µL of the carboxylated PS microparticle solution was used for coating the microscope slides. AFM measurements were performed using a standard NanoWizard IV AFM system (JPK Instruments, Bruker, Billerica, MA). All measurements were conducted in air at 37°C in contact mode using the quantitative imaging technique. In this study, a non-conductive silicon nitride tip (DNP-10, probe D, Bruker, Billerica, MA) was used with an 18 kHz resonance frequency and a 0.06 N/m nominal spring constant. The cantilever spring constants were calibrated using the thermal oscillation method. High-resolution images of carboxylated PS microparticles were captured at a resolution of 128 x 128 pixels, with scanned areas of 40 µm x 40 µm and 20 x 20 µm. A Z-length of 15µm and a set point of 1.796 nN were applied for all images denoted in Figure S1. The JPK data processing software was used to quantify the particle-to-particle distances for each scan size for at least 20 particles. Filters and Parameters such as offset, and multiplier were adjusted accordingly to ensure optimal image quality and accurate representation of surface topography.

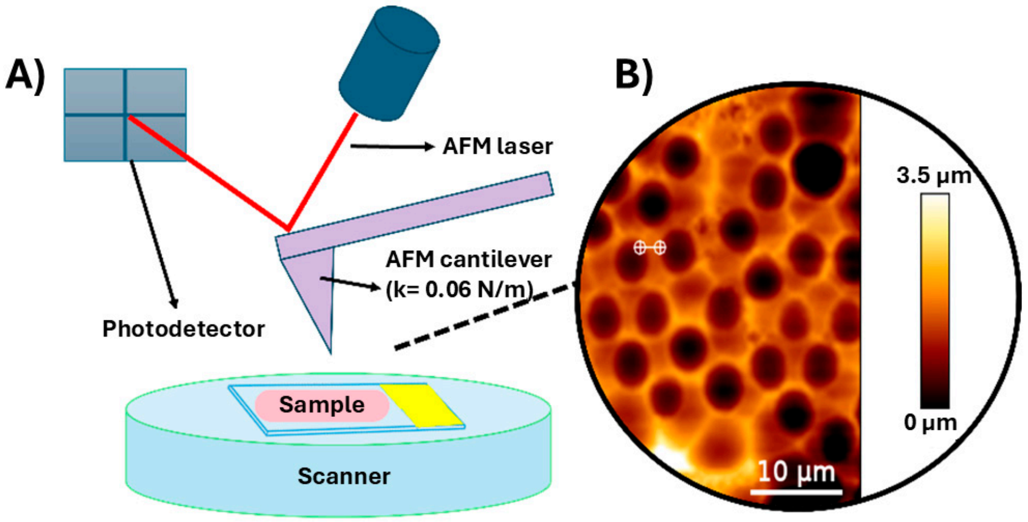


Figure S1: A) Schematic of AFM setup. The cantilever of spring constant k = 0.06 N/m and a laser deflection system to detect surface features were used for AFM operations. B) Representative AFM image showing the topography of carboxylated PS microparticles highlighting the microparticle morphology and inter-particle spacing, with a height scale ranging from 0 to 3.5 µm.

AFM analyzed the topography of cells and microparticles, and the JPK software was used to quantify cellular dimensions and spacing between the carboxylated PS microparticles. This was done to ensure coverage of surfaces by cells or microparticles for accuracy of contact angle measurements. To determine whether the spacing between carboxylated PS microparticles was smaller than the droplet size used in the contact angles measurements, the diameter of the carboxylated PS microparticle was compared to the spacing. A total of 20 particle-to-particle distances were measured in both 20 μm and 40 μm scan areas. Based on the surface topography and analysis using JPK processing software, the average values of spacings were 1.55 ± 0.65 μm within a 400 μm² scan area and 2.50 ± 1.10 μm within a 1600 μm² scan area was determined and illustrated in Figure S2. These values are significantly smaller than the radius of the droplet with a volume of 1.56 mm. These results indicate that the volume of the liquid drop that was used in the contact angle measurements is sufficiently large to cover just cellular or carboxylated PS microparticle surface.

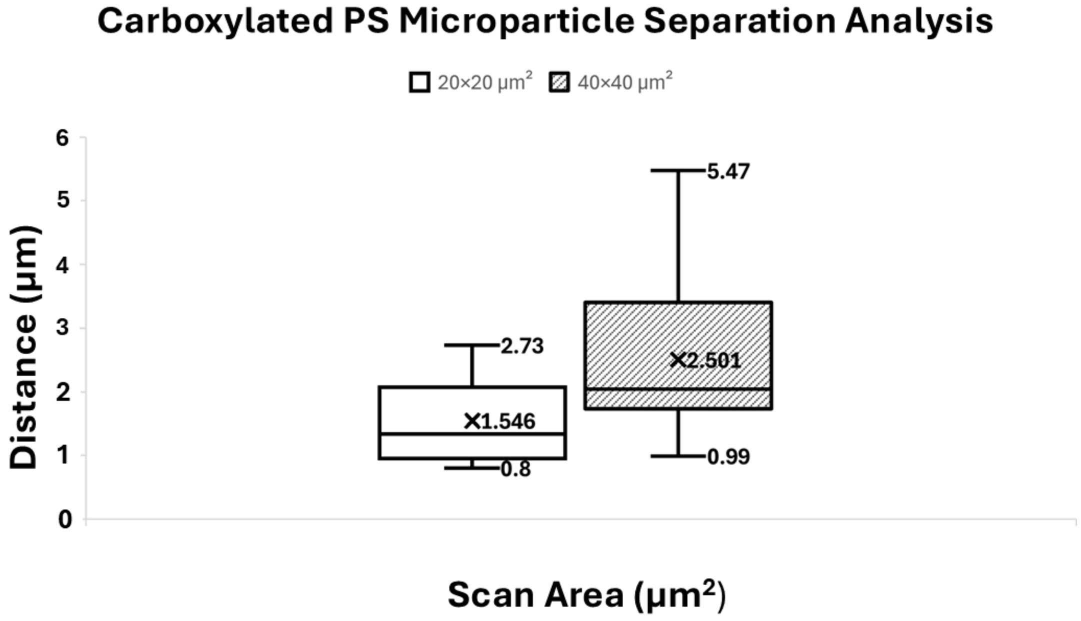


Figure S2. Box plot indicating the distribution of carboxylated PS microparticles measured with AFM within 20 × 20 μm² and 40 × 40 μm² scan areas. The median particle separation of 1.55 μm (range: 0.8–2.73 μm) for the 20 × 20 μm² area and 2.50 μm (range: 0.99–5.47 μm) for the 40 × 40 μm² area is demonstrated based on 20 measurements per scan size.

## S5. Linear Mixed Model Analysis of Contact Angle Data

To assess the factors influencing hChs contact angle measurements, a Linear Mixed Model (LMM) was employed. This approach accounts for both fixed effects (Disease status, Solvent type, Time in culture, and their interactions) and random effects (individual donor variability). The model was fitted to the contact angle data using Jamovi, with Contact Angle as the dependent variable. Tabls S4. summarizes the overall fit of the Linear Mixed Model.

**Table S4. Model Fit Summary**

| Type | $R^2$ | LRT $\chi^2$ | df | P |
|---|---|---|---|---|
| Conditional | 0.886 | 216.926 | 30 | <.001 |
| Marginal | 0.857 | 216.750 | 29 | <.001 |

The conditional $R^2$ indicates that 88.6% of the variance in contact angle is explained by both fixed and random effects. The Marginal $R^2$ shows that 85.7% of the variance is explained by fixed effects alone. The significant p-values (<.001) from the Likelihood Ratio Test (LRT) confirm that the fitted model provides a significantly better fit compared to a null model.

The omnibus tests reveal that Disease status (P = 0.036), Solvent type (P < .001), and Time in culture (P < .001) all have a statistically significant overall effect on the contact angle.

**Table S5. Fixed Effects Omnibus Tests**

| Effect | F | df (res) | df | P |
|---|---|---|---|---|
| Disease | 9.709 | 4.00 | 1 | 0.036 |
| Solvent | 286.940 | 56.00 | 2 | <.001 |
| Time | 5.367 | 56.00 | 4 | <.001 |
| Disease * Solvent | 0.576 | 56.00 | 2 | 0.566 |
| Disease * Time | 2.102 | 56.00 | 4 | 0.093 |
| Solvent * Time | 1.028 | 56.00 | 8 | 0.427 |
| Disease * Solvent * Time | 0.893 | 56.00 | 8 | 0.529 |

Tabls S5. presents the results of omnibus F-tests for each fixed effect and their interactions, indicating their overall statistical significance. The omnibus tests reveal that Disease status (P = 0.036), Solvent type (P < .001), and Time in culture (P < .001) all have a statistically significant overall effect on the contact angle.

**Table S6. Parameter Estimates (Fixed Coefficients)**

| Names | Effect | Estimate | SE | 95% CI Lower | 95% CI Upper | df | t | P |
|---|---|---|---|---|---|---|---|---|
| (Intercept) | (Intercept) | 74.899 | 1.60 | 71.70 | 78.10 | 4.00 | 46.8000 | <.001 |
| Disease 1 | OA-H | 9.980 | 3.20 | 3.57 | 16.39 | 4.00 | 3.1180 | 0.036 |
| Disease1 * Time2 | (OA-H)*(7-0) | 11.578 | 4.66 | 2.26 | 20.90 | 56.00 | 2.4867 | 0.016 |

Table S6. provides the estimated coefficients for the significant fixed effects and interactions. Non-significant interaction terms are not shown for brevity. The (Intercept) of 74.899 mJ/m$^2$ (P < .001) represents the estimated baseline contact angle. OA chondrocytes exhibit a statistically significantly higher contact angle (estimated 9.98 degrees higher) compared to Healthy chondrocytes (P = 0.036). A significant interaction between Disease and Time at the 7-0 time point (P = 0.016) indicates that the difference in contact angle between OA and Healthy groups becomes more pronounced specifically at 7 days in culture.

**Table S7. Random Components**

| Groups | Name | Variance | SD | ICC |
|---|---|---|---|---|
| Donor | (Intercept) | 12.1 | 3.48 | 0.199 |
| Residual | | 48.8 | 6.98 | |

Table S7. quantifies the variability attributed to random effects in the model. Approximately 19.9% of the total variability in contact angle measurements can be attributed to differences between individual donors (ICC = 0.199). This justifies the use of a mixed model, as it effectively accounts for this donor-specific variability.
